# Supplementary material for: Ontology-driven and weakly supervised rare disease identification from clinical notes
Source: BMC Med Inform Decis Mak. 2023 May 5;23:86. doi: 10.1186/s12911-023-02181-9 (PMC10162001; doi:10.1186/s12911-023-02181-9)
Supplement: Supplementary file 1 — Additional file 1. [file 12911_2023_2181_MOESM1_ESM.pdf]

Supplementary material 1 - Further Parameter Settings and Results

Weak Rule Parameter Tuning

The results of parameter tuning for weak labelling rules regarding F_1, recall, and precision scores, are displayed in Table S1-1. We tuned through a grid search the possible values of $p \in \{1e{-}4, 5e{-}4, 1e{-}3, 5e{-}3, 1e{-}2, 5e{-}2, 1e{-}1\}$ and $l \in \{2, 3, 4\}$, and selected the model based on recall and F_1 scores in Text-to-UMLS linking. For MIMIC-III discharge summaries, the results were based on the 400 validation set of manually annotated mention-UMLS pairs. The parameter p controls the corpus-based "prevalence" of the disease concept, which is related to epidemiological information, e.g., the actual prevalence of a rare disease in the cohort. A higher p resulted in more disease concepts selected, thus higher recall but generally less precision. The parameter l controls the mention length, a key threshold to filter out abbreviations, which are usually ambiguous in their meanings. A higher l thus generally resulted in a higher precision but lower recall. We observed that a corpus-based "prevalence" threshold of 0.005 and a mention character length threshold of 3 resulted in the best F_1 score for the dataset. We thus recommend to set $p \in \{0.005, 0.01\}$ and $l \in \{3, 4\}$ and used p as 0.005 and l as 3 for MIMIC-III discharge summaries.

Also, we found that the results were not sensitive to window size, i.e. the input tokens before and after the mention. Thus, all models used the default window size of 5.

Table S1-1 F_1, Precision (P), and Recall (R) scores with respect to the weak rule parameters p and l in Text-to-UMLS linking for MIMIC-III discharge summaries (with the highest F_1 score in bold)

	l = 2			l = 3			l = 4		
	F_1	P	R	F_1	P	R	F_1	P	R
p = 0.0001	73.7%	64.1%	86.6%	59.3%	86.5%	45.1%	64.3%	87.8%	50.7%
p = 0.0005	73.7%	59.6%	96.5%	82.6%	91.5%	75.4%	79.8%	91.0%	71.1%
p = 0.001	72.8%	57.3%	100.0%	80.8%	91.2%	72.5%	79.8%	91.0%	71.1%
p = 0.005	71.5%	55.7%	100.0%	**89.8%**	90.1%	89.4%	87.5%	91.5%	83.8%
p = 0.01	71.0%	55.0%	100.0%	89.7%	87.3%	92.3%	88.5%	90.4%	86.6%
p = 0.05	63.7%	46.7%	100.0%	64.0%	47.0%	100.0%	64.3%	47.3%	100.0%
p = 0.1	60.0%	42.9%	100.0%	60.0%	42.9%	100.0%	60.0%	42.9%	100.0%

Embedding and Fine-tuning Settings

We controlled the same window size (as 5) in baselines with word2vec embeddings and BERT model fine-tuning.

For the word2vec embeddings pre-trained on MIMIC-III discharge summaries, we used Gensim library[1] with Continuous Bag of Words algorithm, without filtering vocabularies by frequency (min_count=0). We experimented with the dimensions of 100, 300, and 768 (see Table 4 in the paper).

For fine-tuning BERT models, we used the average pooling of the mention's sub-tokens representations in the second-last layer (same as the Contextual Mention Representation, with fine-tuning instead of static embedding), followed by a linear layer with softmax activation and cross-entropy loss. The learning rate, warmup steps, and weight decay were

[1]https://radimrehurek.com/gensim/auto_examples/tutorials/run_word2vec.html

5e-05, 500, and 0.01, resp., set up using Huggingface Trainer[2], and trained with 3 epochs. We fine-tuned the BlueBERT-based model (see Table 4 in the paper).

Results on Different Encoding Strategies

The first encoding strategy is *mention masking*, whether or not to mask the mention in the full context window. The intuition behind this is to explore the potential of a language model to confirm a phenotype solely based on the surrounding context but not the mention itself.

The second encoding strategy is *using document structure names* (or template section names) to enhance local context. If the document structure name s is available in the dataset, we add s before the context window t with a separation token [SEP] in between.

Results on the different encoding strategies for Text-to-UMLS linking in MIMIC-III discharge summaries are displayed in Table S1-2. Non-masked encoding achieved better results than masked encoding. Using document structure names further boosted recall scores on the validation and the test set. We used non-masked encoding (with document structure names for MIMIC-III discharge summaries only) for data representation.

Table S1-2 Comparison among encoding strategies for weakly supervised Text-to-UMLS linking on MIMIC-III discharge summaries

	validation set (n=142+/400)			test set (n=187+/673)		
Text to UMLS	P	R	F_1	P	R	F_1
non-M	89.9	87.3	88.6	**81.3**	90.9	**85.9**
non-M+DS	**90.1**	**89.4**	**89.8**	80.4	**92.0**	85.8
M	86.5	63.4	73.2	78.6	61.0	68.7
M+DS	86.4	62.7	72.7	78.0	62.6	69.4

M denotes mention masking and non-M denotes no mention masking applied. DS denotes using document structure names. The non-M+DS model was trained on the full set of weakly labelled data, without tuning the optimal number of data, thus slightly below results in Table 2. BlueBERT-base (PubMed+MIMIC-III) was used to encode the text sequences.

NLP with Strong Supervision vs. ICD for Admission-level Rare Disease Identification

Figure S1-1 shows the results of the NLP pipeline with strong supervision compared to ICD codes for admission-level rare disease phenotyping. The results were generally consistent with the weak supervision approach (in Figure 4 in the paper) that NLP-based results greatly complement the code-based rare disease cohort. Generally, a higher accuracy with a less number of admissions was predicted by strong supervision compared to weak supervision (e.g. the accuracy was 25.5% or 14/55 predicted by “Retinitis Pigmentosa” for strong supervision, compared to 8.2% or 15/183 predicted by weak supervision).

Overall Admission-level and Mention-level Results

Table S1-3 shows the admission-level rare disease phenotyping results for MIMIC-III discharge summaries.

Table S1-4 and S1-5 show the overall mention-level (Text-to-ORDO) and admission-level results of two radiology report datasets in the US (MIMIC-III) and the UK (NHS Tayside). For Tayside data, the recall was lower as we manually identified new rare disease mentions that were not included in the candidate mentions from SemEHR. Weak supervision (WS) achieved better recall than transferring the SS model in results from both Tables.

[2] `https://huggingface.co/docs/transformers/main_classes/trainer`

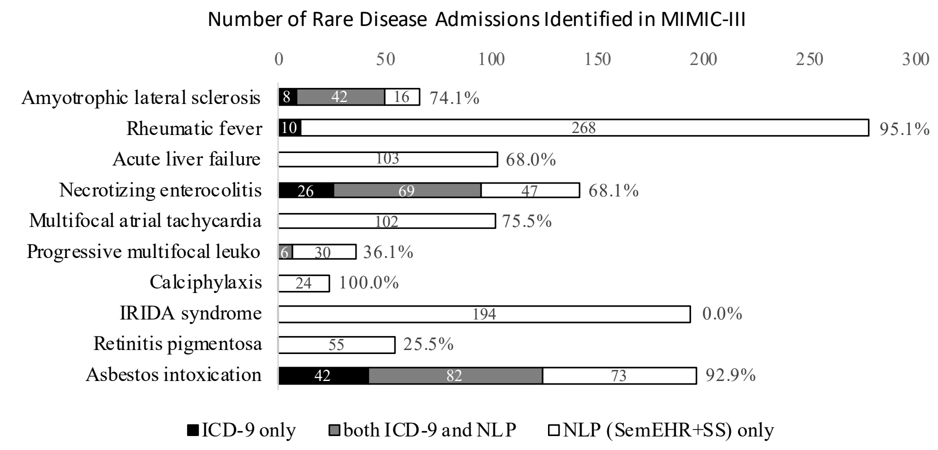

Figure S1-1 Number of rare disease patient stays from MIMIC-III (n=59,652): ICD (code-based) vs. NLP (text-based, with *strong* supervision). The 10 rare diseases are the same as those presented for weak supervision in Figure 4 in the paper. Admissions are split into those *only* identified through links from ICD-9 codes (in black), those *only* identified from free texts with strong supervision (NLP, in white), and the intersection of cases from both ICD-9 and NLP (in grey). The percentage after each horizontal bar shows the accuracy of NLP based on the manual assessment of the identified cases.

The code-based approach (ICD) also did not show an advantage in identifying more rare disease admissions (see recall, R), and overall performance (see F_1), comparing ICD or "ICD ∪ SemEHR+WS" with the (best) SemEHR+WS setting in Table S1-5 and Table S1-3, but the results may be biased towards methods adapting SemEHR as it was used as a starting source to create candidate mentions for the manual annotation.

Table S1-3 Micro-level results of admission-level rare disease identification for MIMIC-III discharge summaries

Admission to ORDO	validation (n=30+/117∗55) P	R	F_1	test (n=42+/192∗82) P	R	F_1
SemEHR	15.4	**93.3**	26.4	12.7	**95.2**	22.3
+ rules	39.2	66.7	49.4	38.9	88.1	54.0
+ WS (rules+BERT)	**57.1**	66.7	**61.5**	**49.3**	78.6	**60.6**
+ SS (anns+BERT)	-	-	-	**61.1**	78.6	**68.7**
ICD	56.2	30.0	39.1	27.3	21.4	24.0
ICD ∪ SemEHR+WS	50.0	70.0	58.3	40.4	85.7	55.0
ICD ∪ SemEHR+SS	-	-	-	45.9	81.0	58.6

The micro-level metric counts each admission and an associated ORDO concept (or an admission-ORDO pair) as a single instance. The column statistics (n=N_++/N_d ∗ N_l) show the number of positive data N_+, admissions (or discharge summaries) N_d, and unique candidate rare diseases (or ORDO concepts) N_l in the dataset. WS, weak supervision; SS, strong supervision; anns, annotations. BlueBERT-base (PubMed+MIMIC-III) was used as the BERT model. ICD denotes the approach to matching ICD-9 codes to ORDO concepts. The union sign (∪) denotes merging and de-duplicating the cases identified from the two methods. Precision (P) and F_1 for ICD-based methods may be lower than actual values, as all candidate mentions were from SemEHR.

Table S1-4 Results on rare disease identification (Text-to-ORDO) for MIMIC-III and Tayside radiology reports

Text to ORDO	MIMIC-III Radiology (n=46+/198) P	R	F_1	Tayside Brain Imaging (n=42+/283) P	R	F_1
SemEHR	22.9	**93.5**	36.8	13.1	**78.6**	22.4
+ WS (transfer)	48.8	84.8	61.9	31.4	76.2	44.4
+ SS (transfer)	86.5	69.6	77.1	**53.2**	59.5	56.2
+ rules (tuned)	84.8	84.8	84.8	31.4	76.2	44.4
+ WS (in-domain)	68.3	89.1	77.4	26.4	**78.6**	39.5
+ WS (+ tuning R)	78.2	**93.5**	85.1	32.4	**78.6**	45.8
+ WS (+ tuning F_1)	**86.7**	84.8	**85.7**	46.3	73.8	**56.9**

The column statistics (n=N_++/N) shows the number of positive data N_+ and the overall number of samples N in the dataset. WS, weak supervision; SS, strong supervision. The original parameters for WS were $p = 0.005$ and $l = 3$. The new parameters for best recall (R) were $p = 0.01$ and $l = 4$ and for best F_1 were $p = 0.0005$ and $l = 4$, for both datasets. For SemEHR+rules, rules were aggregated with an OR operation and $p = 0.0005$ and $l = 4$.

Table S1-5 Micro-level results of admission-level rare disease identification for MIMIC-III and Tayside Radiology Reports

Admission to ORDOs	MIMIC-III Radiology (n=29+/145∗43) P	R	F_1	Tayside Brain Imaging (n=41+/273∗65) P	R	F_1
SemEHR	19.4	**93.1**	32.1	12.8	**78.0**	22.0
+ WS (transfer)	38.7	82.8	52.7	30.7	75.6	43.7
+ SS (transfer)	**83.3**	69.0	75.5	**53.2**	61.0	56.8
+ rules (tuned)	80.0	82.8	81.4	30.7	75.6	43.7
+ WS (in-domain)	59.5	86.2	70.4	25.8	**78.0**	38.8
+ WS (+ tuning R)	71.1	**93.1**	80.6	31.7	**78.0**	45.1
+ WS (+ tuning F_1)	82.8	82.8	**82.8**	46.3	75.6	**57.4**
ICD	46.4	44.8	45.6	-	-	-
ICD ∪ SemEHR+WS	51.9	**93.1**	66.7	-	-	-

The micro-level metric counts each admission and an associated ORDO concept (or an admission-ORDO pair) as a single instance. The column statistics (n=N_++/N_d ∗ N_l) show the number of positive data N_+, the number of admissions (or discharge summaries) N_d, and the number of candidate rare diseases (or ORDO concepts) N_l in the dataset. WS, weak supervision; SS, strong supervision. The original parameters for WS were $p = 0.005$ and $l = 3$. The new parameters for best recall (R) were $p = 0.01$ and $l = 4$ and for best F_1 were $p = 0.0005$ and $l = 4$, for both datasets. For SemEHR+rules, rules were aggregated with an OR operation and $p = 0.0005$ and $l = 4$. The union sign (∪) denotes merging and de-duplicating the cases identified from the two methods. For ICD ∪ SemEHR+WS, the WS model was "in-domain + tuning R", the one re-trained with in-domain data and optimised recall. Precision (P) and F_1 for ICD-based methods may be lower than actual values, as all candidate mentions were from SemEHR.
