# Supplementary material for: Ontology-driven and weakly supervised rare disease identification from clinical notes
Source: BMC Med Inform Decis Mak. 2023 May 5;23:86. doi: 10.1186/s12911-023-02181-9 (PMC10162001; doi:10.1186/s12911-023-02181-9)
Supplement: Supplementary file 2 — Additional file 2. [file 12911_2023_2181_MOESM2_ESM.pdf]

Supplementary material 2 - Examples of Rare Disease Identification and Ontology Matching

Examples of Rare Disease Text Phenotyping

Table S2-1 (on page 2) shows some selected prediction errors and a few correct predictions. The first four examples are the false positives selected in the evaluation data for the weak supervision model due to semantic type errors, hypothetical contexts, or other issues. The last five examples are those selected from the identified rare disease cohort for Retinitis Pigmentosa and Rheumatic Fever. Synonyms in UMLS could help identify some name variations, e.g. “tracheobronchomalacia” for Williams-Campbell syndrome, and “acute rheumatic fever” for Rheumatic fever, but also introduces false positives especially regarding abbreviations, e.g. “EMA” and “RP”. The complex context in the clinical notes, including the relative’s diseases or hypothetical mentions, although only representing a small part of cases, were still challenging for the NLP pipeline (SemEHR+WS), as these were not explicitly considered in the weakly supervised training process. We also note that there were errors in parsing the document structure name through regular expressions in SemEHR, which might affect the predictions.

Ontology Matching from ORDO to ICD-9

Table S2-2 (on page 3) shows 10 examples of rare disease concepts and their ontology matching from ORDO to UMLS, ICD-10, and ICD-9. The rare diseases are the same as those presented in Figure 4 in the paper and Figure S1-1 in Supplementary material 1.

Table S2-1 Examples of wrong and correct rare diseases identified by SemEHR with the weak supervised phenotype confirmation model from MIMIC-III discharge summaries

ROW_ID	Document Structure	Text (with **mention** in bold)	UMLS	ORDO	Pred	Label	Potential Reason
26825	pertinent_results (should be pathology)	Pathology: ...Immunostains for cytokeratin AE1/3 and CAM 5.2, CD-68, CD-79a, CD-138, S-100, LCA absorbed CEA, **EMA**, CD34, CD31, TTF-1, actin, desmin, MNF-116, calcitonin, and thyroglobulin are negative...	C0268596	26791 Multiple acyl-CoA dehydrogenase deficiency	T	F	negation with a long context, ambiguous mention (EMA as epithelial membrane antigen), and semantic type error (negative test)
869	Hospital_course	Brief Hospital Course: ## Dyspnea - ...DFA for flu was negative; urinary **legionella** antigen was also negative...	C0023241	549 Legionellosis	T	F	semantic type error with negation (negative test)
8960	History_of_Past_Illness	Past Medical History: 1. Diagnosed in his early years with bilateral uveitis, clinically had bilateral uveitis significant with loss of vision and **sarcoid** floaters in both eyes. . .	C0036202	797 Sarcoidosis	T	F	not enough information (sarcoid floater not necessary means sarcoid)
46361	pertinent_results (should be impression)	IMPRESSION: ...Of note prior chest CT scans have findings suggesting a propensity to **tracheobronchomalacia**, as well as moderately severe emphysema....	C0340231	411501 Williams-Campbell syndrome	T	F	hypothetical context
48161	Admission_Medications	Medications on Admission: ...Vitamin A palmitate 100,000 units 1.5 tablets dialy for **retinitis pigmentosa**, acetaminophen, tums, Mylanta, OTC Prilosec prn	C0035334	791 Retinitis Pigmentosa	T	T	correct
26351	Hospital_course	...Her ASA continued to be held due to the **RP** bleed but was restarted after 48 hrs of stable Hct. . .	C0035334	791 Retinitis Pigmentosa	T	F	ambiguous abbreviation (Retroperitoneal bleeding)
12659	History_of_Past_Illness	Past Medical History: PMHx: ... 7. h/o of **rheumatic fever** with Sydenham's chorea. . .	C0035436	3099 Rheumatic fever	T	T	correct
20984	Hospital_course	The patient never reported any pharyngitis, but given his complaints of diffuse arthralgias, myalgias, migrating neuropathic pain, there was some concern of **rheumatic fever**, as the patient had 2 ASO screens performed which were both negative.	C0035436	3099 Rheumatic fever	T	F	hypothetical context
11568	basic (should be family history)	FAMILY HISTORY: ...2) His mother has an enlarged heart which may be secondary to a history of **acute rheumatic fever**...	C0035436	3099 Rheumatic fever	T	F	a relative's disease

Prediction errors are coloured with red in "Pred" (third-last) column. For columns "Pred" and "Label", "T" means that the prediction or gold is *True* and "F" indicates *False.* The wrongly parsed document structure names in the second column are marked with corrected ones in the form of "(should be XXX)".

Table S2-2 Ontology concept matching among ORDO, UMLS, ICD-10, and ICD-9 based on publicly available sources

ORDO	ORDO Preferred Label	UMLS	ICD-10	ICD-9-NZ (from ICD-10)	Preferred Label	ICD-9-BP (from UMLS)	Preferred Label
803	Amyotrophic lateral sclerosis	C0002736	$<$G122	-	-	335.20	Amyotrophic lateral sclerosis
3099	Rheumatic fever	C0035436	$>$I011, $>$I00, $>$I010, $>$I012, $>$I018, $>$I019	3911, 390, 3910, 3912, 3918, 3919	Acute rheumatic endocarditis; Rheumatic fever without mention of heart involvement; Acute rheumatic pericarditis; Acute rheumatic myocarditis; Other acute rheumatic heart disease; Acute rheumatic heart disease, unspecified	390-392.99	ACUTE RHEUMATIC FEVER
90062	Acute liver failure	C0162557	$<$K720	-	-	-	-
391673	Necrotizing enterocolitis	C0520459	$=$P77	7775	Necrotizing enterocolitis in newborn	-	-
3282	Multifocal atrial tachycardia	C0221158	$<$I471	-	-	-	-
217260	Progressive multifocal leukoencephalopathy	C0023524	$=$A812	0463	Progressive multifocal leukoencephalopathy	46.3	Progressive multifocal leukoencephalopathy
280062	Calciphylaxis	C0006666	$<$E835	-	-	-	-
791	Retinitis pigmentosa	C0035334	$<$H355	-	-	-	-
209981	IRIDA syndrome	C0085576	$<$D508	-	-	-	-
2302	Asbestos intoxication	C0003949	$<$J61	-	-	501	Asbestosis

$=$, $>$, and $<$ in ORDO-to-ICD-10 mappings (all from ORDO) indicate exact, broader-to-narrower, and narrower-to-broader matching, respectively. Narrower-to-broader matching ($<$) from ORDO to ICD-10 was not used for phenotyping, as it may result in common or non-rare diseases' ICD codes. All ORDO-to-UMLS mappings (all from ORDO) indicate exact matching ($=$). "ICD-9-NZ" denotes the set of ICD-9 codes linked from ICD-10 codes using the matching from the Ministry of Health, New Zealand, `https://www.health.govt.nz/nz-health-statistics/data-references/mapping-tools/mapping-between-icd-10-and-icd-9`. "ICD-9-BP" refers to the set of ICD-9 codes linked from UMLS based on the ICD-9-CM ontology (version 2020AB) in BioPortal, `https://bioportal.bioontology.org/ontologies/ICD9CM`.
